# Mediterranean Journal of Rheumatology June 2019 Highlights

**DOI:** 10.31138/mjr.30.2.84

**Published:** 2019-06-29

**Authors:** Dimitrios P. Bogdanos

**Affiliations:** Department of Rheumatology and Clinical Immunology, Faculty of Medicine, School of Health Sciences, University of Thessaly, Larissa, Greece

This issue of the Journal includes papers of great interest.

Nicola Bizzaro, in his expert opinion article,^1^ critically discusses the evolving role of the newly developed analytical profiling technologies for autoantibody diagnostics. These systems, which are based on micronized components (protein chips or arrays), consist of solid phase-linked autoantigens capable of simultaneously detecting many autoantibodies at the same time. Their *pros* and *cons* are considered, and unresolved questions related to the pre- and post-analytical phases are raised.

A joint effort by Greek rheumatologists and cardiologists led to a position paper on the emerging role of screening and early diagnosis of pulmonary arterial hypertension in connective tissue disorders. Based on bibliographic data and clinical experience obtained over the years, Demerouti et al.^2^ issued ten key commandments for the Greek (and not only) rheumatologist regarding key clinical issues for the management of pulmonary arterial hypertension that the Rheumatologist should be alert of.

Heidari et al.^3^ review epidemiological, clinical and immunological data on the role of vitamin D deficiency in rheumatoid arthritis. Vitamin D is a principal immuno-regulator and has been shown to be a key player in the development of RA. As the authors discuss, clinical challenges are still emerging in relation to vitamin D intake and RA as the data are conflicting and is not clear which dose of vitamin D provides more benefit, if any.

Katerina Chatzidionysiou^4^ assesses the close relationship between rheumatic disease and art created by great painters suffering from diverse rheumatological conditions. As the author points out “the disease was in many cases the force that led to change in style and technique that contributed substantially to their establishment as great masters, supporting the fact that some of the greatest, most beautiful art is born of great suffering”.

In a cross-sectional study involving 111 Iranian patients with rheumatoid arthritis, Anari et al.^5^ compared the diagnostic value of conventional radiography (CR) versus ultrasonography but failed to find a significant difference between these two methods in the detection of bone erosions. The authors plan a study to be conducted in a larger cohort, which is highly anticipated. Small sample numbers may preclude the findings from being extrapolated, whereas larger sample sizes may intensify the detection of differences, emphasizing statistical differences that could be of clinical relevance.

An Egyptian group of investigators assessed the genetic association between Growth Differentiation Factor 5 (GDF5) gene (rs143383T/C) single nucleotide polymorphism (SNP), one of the most important osteoarthritis risk allele, in a group of Egyptian primary knee osteoarthritis patients.^6^ The study failed to reveal a significant association. Because of the limited number of patients assessed, it is difficult to understand whether the lack of genetic association is true or is epiphenomenal.

An informative case study, by Gorial et al.^7^ describes a six-year-old boy, pre-diagnosed with galactosemia, who developed musculoskeletal manifestations.

The issue also includes a research protocol. O’Brien et al.^8^ will be the first to define RA-specific accelerometer cut-points, and to validate the ActiGraph accelerometer and activPAL^μTM^, for the measurement of sedentary time and physical activity in RA.

In a letter to the Editor, Kontaxi et al.^9^ describe a case of a 5year old girl, the youngest survivor of severe acute respiratory distress syndrome, a potentially life-threatening emergency condition with a very short disease onset and a rapidly progressing pneumonitis that led to the diagnosis of childhood onset systemic lupus erythematous. The case is of great interest as it offers a significant body of knowledge in the respective field, which can assist efforts to enhance our clinical knowledge and to improve clinical performance, and practice behaviour.

Enjoy the reading.
